# An Investigation into the Prevalence of Enamel Hypoplasia in an Urban Area Based on the Types and Affected Teeth

**DOI:** 10.3390/children11040474

**Published:** 2024-04-15

**Authors:** Valbona Disha, Marin Zaimi, Elizana Petrela, Fatbardha Aliaj

**Affiliations:** 1 Department of Dentistry, Faculty of Medical Science, Albanian University, 1001 Tirana, Albania; v.disha@albanianuniversity.edu.al; 2 Department of General and Visceral Surgery, Ulm University Hospital, 89075 Ulm, Germany; 3 Faculty of Medicine, Head of Statistic Service, UHC “Mother Teresa”, University of Medicine Tirana, 1001 Tirana, Albania; 4 O.SH.K.SH, 1001 Tirana, Albania

**Keywords:** enamel hypoplasia, types, permanent teeth, factors, prevalence

## Abstract

Enamel hypoplasia (EH) is a qualitative defect, and it can have a significant impact on oral health. The aim of this study was to evaluate the prevalence of enamel hypoplasia in urban area in Albania. Methodology: In total, 234 children of both sexes aged 8–12 years old were randomly selected in five schools in Tirana, Albania. They underwent an intra-oral examination. Diagnostic criteria were in accordance with a European meeting on MIH held in Athens, 2003, and the FDI. Medical history was retrieved using questionnaires, and data obtained from clinical examination were recorded. Results: The prevalence of enamel hypoplasia was 12.8%. The most commonly occurring enamel hypoplasia was the mild type (58.62%). The mandibular first molar showed the highest prevalence of enamel hypoplasia (19.5%), and the maxillary canines and premolars were the least affected (2.3%). In this study, medical story did not have a significant effect on enamel hypoplasia. Conclusions: The prevalence of enamel hypoplasia remains high at 12.8%. Interestingly, the features of enamel hypoplasia were consistent across both sexes, with no correlation found between them. The predominant occurrence of mild enamel hypoplasia underscores the importance of implementing oral hygiene strategies in schools to mitigate its progression.

## 1. Introduction

Development defects of enamel (DDE) are alterations resulting from early damage to the enamel organ during the process of odontogenesis. Disturbances during enamel development result in macroscopic and structural changes in it [1]. They may be quantitative or qualitative in nature. Enamel hypoplasia (EH) is a qualitative defect that occurs during the secretion phase. It is characterized by a reduction in enamel thickness, and may vary according to clinical features [2,3]. Due to enamel defects, enamel hypoplasia has a higher predisposition to dental caries compared with enamel defects that are associated with opacities. Hypomineralization (MIH) is another enamel defect (qualitative defect) that occurs during the maturation phase, and may vary according to clinical features from the mild form (opacity) to post-eruptive breakdown. In these cases, differences between enamel hypoplasia and post-eruptive breakdown of the enamel are difficult to determine. Both forms of enamel defects (ED) can have a significant impact on oral health [4,5].

Enamel defects may appear in both dentitions and temporary and permanent teeth. Due to the defects of the teeth’s surface in both cases, like enamel hypoplasia and MIH with a post-eruptive breakdown of the enamel, teeth become more sensitive, increasing the risk for caries and increasing pain, which have an impact on patients’ daily routines, affecting their quality of life.

Enamel hypoplasia is a frequently observed defect in dental practice, and it is currently an important issue. Even though the etiology of hypoplasia is not completely clear, multiple factors during pre-, peri- and post-natal periods have been associated with enamel hypoplasia. Genetic and hereditary factors such as amelogenesis imperfecta may be involved, along with systemic and environmental factors such as fluoride intake, medications, nutritional deficiencies, prenatal infections, chicken pox or other early childhood disease, and low vitamin D serum levels in both mother and child [5,6,7,8,9,10,11,12]. Studies have shown that low-birth-weight preterm infants presented a higher prevalence of hypoplasia than did normal-birth-weight controls, and the primary teeth most affected by hypoplasia were maxillary incisors [13].

Enamel hypoplasia can make children prone to dental hypersensitivity. As a consequence, it may cause them to eat on the healthy side of the mouth (therefore causing the fast formation of dental plaque on the side where teeth are affected by enamel hypoplasia), cause insufficient tooth brushing, increase the risk of caries development, and cause difficulty in achieving effective local anesthesia [14,15]. On one hand, hypoplastic areas are reported to be highly susceptible to dental caries and tooth wear [16,17,18]; on the other hand, the restoration of these teeth is difficult because sensitivity and pain reduce the cooperation of children and these teeth need new restoration because of repetitive failure [4,5,17].

Different studies have shown that the prevalence of enamel hypoplasia has increased [19,20]. The average worldwide prevalence of hypoplasia from one study published in 2018 was 13.1% [21]. In Albania, the only study [22] for MIH prevalence, which reported it to be 14%, was limited in terms of it taking data only on incisors and first molars. There are no data in the literature on the prevalence of enamel hypoplasia in Albania.

The presence of EH has a direct effect on quality of life. It appears on the surface of teeth, giving an anesthetic view. The presence of development defects of the enamel affects the emotional state of children [23] and may affect their daily life [24]. EH treatment varies according to its appearance. Conservative treatment is the most recommended treatment in children, but restorative treatment may take place when the esthetics are concerning [25]. In Albania, no epidemiologic study has analyzed clinical features of enamel hypoplasia.

General information about oral health is very important to positively change children’s habits toward oral hygiene. All preventive techniques in dentistry are effective when patients appreciate their oral health. Recent studies [26,27] have shown that the institutions that implement oral health programs take greater care for oral hygiene. On the other hand, Alessandro et al. [28] showed that the presence of enamel defects may affect the psychosocial behavior of children toward oral hygiene at home. Enamel hypoplasia manifests clinically with defective and retentive areas, facilitating the accumulation of dental plaque. For these reasons, bacterial biofilm accumulates on all teeth, making them susceptible to caries; this even happens on teeth without enamel defects [29]. Moreover, oral hygiene care does not seem to be the same between sexes. According to a study [30], females were more careful about oral hygiene than males were. Since schools in Albania have dental clinics within their premises, the implementation of new strategies for increasing the motivation of children to improve their oral hygiene is very important.

Also, regarding the appearance of hypoplasia in relation to sex, there are different opinions. In one study [31], a higher prevalence of EH in males was reported, while in another study [12], the opposite results were reported. On the other hand, others [22] did not find any significant differences between sexes.

As there are no existing Albanian studies on the prevalence of enamel hypoplasia (EH), our research aims to fill this gap by conducting cross-sectional studies. Cross-sectional studies are valuable for measuring the prevalence of a health condition within a population at a specific point in time. Through our research, we aim to offer valuable insights into the prevalence and characteristics of enamel hypoplasia among individuals in Albania.

## 2. Materials and Methods

A cross-sectional clinical evaluation of 234 volunteer children aged 8–12 years was carried out from January 2024 to March 2024 in five schools in Tirana, and these children were randomly selected. Ethical approval to conduct the study was obtained from the Council of Ethics UMT, No. 3649/6. This study included a clinical examination of the children as well as the completion of a questionnaire by their parents after they signed a consent form.

The inclusion criteria:

This study included healthy male and female children aged 8 to 12 years who were present on the day of examination and had signed consent forms. All erupted and partially erupted permanent teeth were examined.

The exclusion criteria:

The exclusion criteria comprised children who refused to participate, those with present or past systemic disease, children with fixed orthodontic appliances, those with heavily restored and severely decayed teeth, and children with teeth with enamel defects measuring less than 1 mm in diameter; children from whom parental consent was not obtained were not included in the study.

Data collection:

A clinical examination of children was conducted at the clinics of their schools early in the morning under artificial light. The teeth were examined when wet after they were cleaned with cotton rolls. During intra-oral examination, a mirror and an explorer were used, and dental surfaces were examined to verify the presence of enamel defects. All evaluations were performed only by one trained examiner with 14 years of experience. The examiner was assisted by an assistant who recorded all the data.

During the intra-oral examination, all the dental surfaces of each tooth were examined for enamel defects. Diagnostic criteria were in accordance with the European meeting on MIH held in Athens, 2003 [32] (1. absence or presence of demarcated opacities; 2. post-eruptive enamel breakdown; 3. atypical restorations; 4. extraction of molars due to MIH; 5. failure of eruption of a molar or incisor).

The teeth were examined for enamel hypoplasia. A clinical diagnosis of EH was made, and teeth showing deficiencies in enamel formation were classified according to clinical features (FDI 1992) [33] as mild (pits), moderate (grooves), and severe forms (missing enamel) (refer to Table 1). The presence of EH was documented for each tooth.

All permanent teeth with extensive restorations, severe decay, and enamel defects smaller than 1 mm in diameter were excluded from the examination. Additionally, primary teeth and other enamel defects were also excluded.

Parents completed a questionnaire during the dental examination, which consisted of two parts:

General Information:

- The child’s details such as age, gender, date and place of birth, and current place of residence;
- Information about the parents including their education level and occupation.

Medical History:

- Details regarding the child’s mother’s health during pregnancy, including any diseases and medications taken.
- Information about the type of birth (natural or via operation), whether it was within the normal range or premature, and whether there were any complications during birth. Additionally, the birth weight of the child was recorded.
- Details about the child’s feeding after birth, including whether or not they were breastfed, the duration of breastfeeding, and if they were fed with milk formula.
- The child’s health status during the first 5 years of life, any medications taken during this period, and any current diseases or medical conditions.

The parents of the children participating in the study were provided with informed consent documents, ensuring that they understood the purpose and procedures of the research. They were also assured of confidentiality and anonymity regarding their personal information and that of their children. These measures were taken to uphold ethical standards and protect the privacy of the participants.

Data analyses:

Data analysis was conducted using SPSS 26.0 (Statistical Package for Social Sciences, version 26). For categorical variables (including binary/dichotomous and ordinal scales), frequencies and percentages were calculated. For numerical variables, measures of central tendency and dispersion were determined. Mean and standard deviation were computed for normally distributed data, while median and interquartile range were calculated for non-normally distributed data. Group comparisons were conducted using the Mann–Whitney U test and Chi-square test. *p*-values of *p* < 0.05 were considered statistically significant.

## 3. Results

In total, 243 children participated in the study, comprising 133 females (54.7%) and 110 males (45.3%). The average age of all children was 10.2 ± 1.2 years old, while children with hypoplasia were of an average age of 10.4 ± 1.1 years old. The prevalence of enamel hypoplasia was found to be 12.8% (*n* = 31). The analysis of the distribution of enamel hypoplasia, shown in Table 2, revealed no significant difference between sexes.

Table 3 illustrates the distribution of permanent teeth affected by enamel hypoplasia between sexes. The results indicate a higher prevalence of enamel hypoplasia in males, accounting for approximately 61% of affected teeth, compared with 39% in females. Moreover, enamel hypoplasia was most commonly observed in the first molars. Tooth #46 exhibited the highest prevalence at 19.5%, followed by tooth #16 (16%), #26 (13.8%), #36 (12.6%), #21 (10.3%), and #11 (8%). Conversely, the least affected teeth were the lateral incisors, canines, and premolars, each accounting for 2.3% of cases. Based on the results shown in Table 3, the upper jaw seems to be more affected by enamel hypoplasia compared with the lower jaw.

During the examination of the children, clinical features of enamel hypoplasia (FDI) [33] were diagnosed (Table 4). The mild form of hypoplasia was present at the greatest extent at about 58.6%, the medium form was present in 16% patients, and the severe form was present in 5.7%. Regarding sex, there were no statistically significant differences between forms of hypoplasia except for the moderate form (*p* = 0.001).

The educational backgrounds of parents exhibited variation, as indicated in Table 5. The majority of parents possessed postsecondary or secondary education, with some having attained elementary education. Only two parents did not provide a response. Statistical analysis revealed no significant disparity in the educational levels between mothers of female and male patients (*p* = 0.342), or between fathers of female and male patients (*p* = 0.449).

In children with enamel hypoplasia (as shown in Table 6), the majority of parents had postsecondary education, while the remaining had secondary education. Statistical analysis revealed no significant difference in the educational levels of mothers (*p* = 0.825) and fathers (*p* = 0.961) between female and male patients.

In the parental questionnaires regarding childbirth details, the authors collected data on the birthing process and timing for 243 cases. Of these, 56.4% of births occurred naturally, while 43.6% occurred via surgical delivery (refer to Table 7). Remarkably, 92% of births took place within the expected gestational period, with only 7.4% experiencing complications during delivery. Significantly, among both women and men, cases of normal birth (*p* = 0.01), births within the normal gestational range (*p* < 0.001), and births without complications (*p* < 0.001) predominated.

Among children with hypoplasia, one male and one female experienced complications during birth, although both were born within the predicted timeframe. The average birth weight was 3.32 kg for females and 3.41 kg for males. No child was born underweight, and no correlation was observed between birth weight and the presence of hypoplasia. Refer to Table 7 and Table 8 for the average birth weights of all participating children and those with hypoplasia, respectively.

Breastfeeding was the primary method of feeding for the majority of children (see Table 7 and Table 8), while the remaining were fed with formula milk. The duration of breastfeeding varied among children, ranging from a minimum of 2 weeks to a maximum of 5 years. Interestingly, the average duration of breastfeeding was similar for both genders. Male children were breastfed for a minimum of 4 months to a maximum of 4 years, whereas female children were breastfed for a minimum of 3 months to a maximum of 2 years.

Among the mothers who participated in this study, only a few reported experiencing illnesses during lactation, and very few took medications during this period (refer to Table 7). Interestingly, mothers of children with hypoplasia reported no illnesses and did not require medication during lactation.

Overall, the health condition of the children was generally good. Cases of illnesses during early childhood and current diseases are outlined in Table 7. Among the 15 girls affected by hypoplasia, only 2 had allergies and received treatment. Similarly, among the 16 males with hypoplasia, 1 had ASLO, and another experienced chronic infections necessitating multiple courses of antibiotics.

## 4. Discussion

Enamel hypoplasia continues to be an important discussion in dentistry, because of its prevalence. This cross-sectional study examined the prevalence of EH, its correlation with sex, the most affected teeth, and clinical features. Enamel defects may cause many problems in children, such an increased risk of caries and increasing sensibility of teeth, and may cause esthetic and occlusal problems. Thus, the effective management of EH involves early examination and an understanding of causative factors to minimize lesion progression.

In this study, 12.8% of subjects had enamel hypoplasia, which is lower than the 18.2% reported by Fotedar S. et al. [34], and higher than the 9.2% reported by Warwar AN. et al. [35] and the 3.4% reported by Agrawal A. et al. [36]. Even though it is not possible to compare the results of different studies because of the differences in classification methods and examination techniques, the results of this study revealed that enamel hypoplasia was still a very common oral health problem among children.

The most common form of enamel hypoplasia in this study was the mild type at 58.6%, and only 7.1% of cases belonged to the severe form. Other studies [37,38] that have analyzed enamel defects have revealed that demarcated opacity, the mild form of enamel defects, was the most common. Even though EH prevalence is high, since it is present in most cases as the mild form, these are good findings, because early examination, the implementation of oral hygiene, and other preventive techniques can stop the progress of these lesions.

In the present study, mandibular first molar #46 showed the highest prevalence (19.5%) followed by maxillar first molars #16 (16%) and maxillary first incisors #11 (8%). The least number of hypoplastic teeth involved were the maxillary canines and premolars (2.3%). In another study [35], the maxillary central incisors showed the highest prevalence. On the other hand, the only study for MIH prevalence in Albania [22] revealed that tooth number 36 was the most affected molar and tooth number 32 the least affected incisor.

In this study, statistically significant difference in the prevalence of enamel hypoplasia between males and females was found. This was also reported by Daneshkazemi et al. [39], while Basha S. et al. [37] reported a higher prevalence of enamel hypoplasia among males. In the present study, males showed a greater number of teeth with hypoplasia and they had more than one type of hypoplasia. In the above context, these may be results of the fact that boys have greater nutritional requirements due to them having more rapid growth [37]. Other studies [12] reported that females were more affected than were males. These findings could be due to the earlier eruption of teeth [40] and an increased period of breastfeeding [23].

The average age of the children participating in the study was 10 years, and the average age for children with hypoplasia was 10.4 years. The prevalence of enamel hypoplasia was lower at 8–9 years of age when compared with that at 10–12 years. This was also observed by Fotedar S. et al. [34]. The children who showed the severe form of hypoplasia, comprising 60% of the sample, belonged to the age group of 11 years. The same results were noticed in some other studies [34,41,42] where the prevalence of more diffuse forms of hypoplasia was observed in the older age groups selected. This may have been due to a high exposure to etiological factors over a longer period of time.

In the present study, there was no significant difference in the prevalence of enamel hypoplasia in terms of the parent’s education and occupation, which was the same as what was observed by Cruvinel VR. et al. [43], whereas Ford et al. [17] did not reveal similar findings.

Enamel hypoplasia in the present study was not associated with illness during pregnancy and maternal use of medication. Two recent reviews found that the presence of maternal illness during pregnancy and the use of antibiotics was associated with 40% higher odds of MIH [20,44]. Use of antibiotics was associated with MIH in one study but not maternal illness [45].

In the present study, health issues during birth such as early birth, premature birth, and low weight did not have a significant effect on enamel hypoplasia. In addition, the duration of breastfeeding was not associated with enamel hypoplasia, which is consistent with the findings of recent studies [20,44,46].

Moreover, this study showed that health problems in early childhood did not affect the prevalence of enamel hypoplasia. Jälevik B. et al. [31] revealed similar findings in one study on the development defects of the enamel.

Furthermore, our findings indicate no significant association between medical history and enamel hypoplasia. Nevertheless, it remains imperative to address factors related to hypoplasia.

It is crucial to emphasize that oral health is crucial for individuals’ ability to eat, speak, and express themselves confidently through facial expressions, without experiencing discomfort or disease in the craniofacial complex [47]. Inadequate oral health can significantly impact overall health and contribute to the experience of pain, affecting essential activities such as eating, chewing, smiling, and communication [48]. Oral health obviously has consequences in oral diseases in daily life in terms of physical, psychosocial, and social well-being [49,50]. Numerous studies have shown correlations between poor oral health and various physical health issues such as cardiovascular diseases, diabetes, respiratory infections, and adverse pregnancy outcomes [49]. Additionally, the psychosocial impacts of oral health problems include effects on self-esteem, confidence, and quality of life [50]. Socially, individuals with poor oral health may experience stigma, discrimination, and barriers to social interaction and employment opportunities [50]. Therefore, addressing oral health is crucial not only to maintaining physical health but also for promoting overall well-being and social inclusion. Quality of life research helps estimate the extent of illness burden and identifies priority groups for public health interventions. It also assists in establishing outcome measures for initiatives promoting oral health [51].

Enamel defects indeed carry significant clinical significance and directly relate to public oral health strategies. These defects can serve as indicators of past or ongoing disturbances during tooth development, providing valuable insights into individuals’ oral health status and potential risks for future dental problems. Understanding the prevalence, causes, and impact of enamel defects can inform public health policies and interventions aimed at promoting oral health and preventing dental diseases. By addressing factors contributing to enamel defects, such as nutritional deficiencies, systemic illnesses, or environmental factors, public oral health strategies can effectively target prevention efforts and improve overall dental health outcomes in populations.

The strengths of the study include the fact that the examination of children was conducted only by one trained and experienced examiner, reducing errors during clinical examination.

Thee limitation of the study is that the data regarding medical history were collected through a self-administered parent questionnaire, which may have introduced bias into the information provided.

## 5. Conclusions

The prevalence of enamel hypoplasia remains high at 12.8%. Interestingly, the features of enamel hypoplasia were consistent across both sexes, with no correlation found between them.

The predominant occurrence of mild enamel hypoplasia underscores the importance of implementing oral hygiene strategies in schools to mitigate its progression.

## Figures and Tables

**Table 1:** Clinical features of enamel hypoplasia according to FDI 1992.

Forms of Enamel Hypoplasia	Clinical Features	Teeth (According to Their Symbol)
Mild	Pits (single/multiple, shallow/deep tiny areas of enamel loss.	
Moderate	Grooves or lines of enamel loss (<2 mm wide)	
Severe	Areas of partial or complete absence of enamel of a tooth crown.	

**Table 2:** The distribution of enamel hypoplasia by sex.

Children	Total *n* = 243 (100.0)	Female *n* = 133 (54.7)	Male *n* = 110 (45.3)	*p*-Value *
With hypoplasia	31 (12.8)	15 (11.3)	16 (14.5)	0.231
Without hypoplasia	212 (87.2)	118 (89.7)	94 (85.5)	

* Chi-square test. The percentages are calculated in columns.

**Table 3:** Distribution of permanent teeth affected by enamel hypoplasia.

Teeth	Female (Number of Teeth Affected)	Male (Number of Teeth Affected)	Total
# 11	4	3	7
# 12	1	1	2
# 13	1	1	2
# 14	1	-	1
# 15	-	2	2
# 16	4	10	14
# 21	6	3	9
# 22	-	-	-
# 23	1	-	1
# 24	-	1	1
# 25	-	-	-
# 26	3	9	12
# 31	1	1	2
# 32	1	1	2
# 33	1	-	1
# 34	-	-	-
# 35	-	1	1
# 36	3	8	11
# 41	1	1	2
# 42	-	-	-
# 43	-	-	-
# 44	-	-	-
# 45	-	-	-
# 46	6	11	17
No Total	34	53	87

Mann–Whitney *U*-test: *p* = 0.091.

**Table 4:** The distribution of enamel hypoplasia according to severity level.

Types of Enamel Hypoplasia	Total *n* (%)	Female *n* (%)	Male *n* (%)	Value *p* *
Mild	51 (58.6)	22 (64.7)	29 (54.7)	0.063
Moderate	14 (16.1)	3 (8.8)	11 (20.8)	0.001
Severe	5 (5.7)	2 (5.9)	3 (5.7)	0.356
Atypical restorations	17 (19.5)	7 (20.6)	10 (18.9)	0.117
Total	87 (100.0)	34 (100.0)	53 (100.0)	-

* Chi-square. The percentages are calculated in columns.

**Table 5:** Education of all children’s parents who participated in this study.

Education of Parents	Female (%)		Male (%)	
	Mother *n* = 133	Father = 133	Mother *n* = 110	Father *n* = 110
Elementary education	-	-	1 (0.9)	1 (0.9)
Secondary education	36 (27.0)	51 (38.3)	36 (32.7)	46 (41.8)
Postsecondary education	96 (72.2)	81 (60.9)	73 (66.4)	62 (56.4)
No answer	1 (0.8)	1 (0.8)	-	1 (0.9)

The percentages are calculated in columns.

**Table 6:** Education of parents of children with hypoplasia.

Education of Parents	Female (%)		Male (%)	
	Mother *n* = 15	Father *n* = 15	Mother *n* = 16	Father *n* = 16
Elementary education	-	-	-	-
Secondary education	4 (26.6)	6 (40.0)	4 (25.0)	7 (43.7)
Postsecondary education	10 (66.7)	8 (53.3)	12 (75.0)	9 (56.3)
No answer	1 (6.7)	1 (6.7)	-	-

The percentages are calculated in columns.

**Table 7:** General information from birth to the early childhood period for all children who participated in this study, based on the questionnaire filled in by their parents.

	Total *n* = 243 (%)	Female *n* = 133 (%)	Male *n* = 110 (%)	*p*-Value *
Natural birth/operation	137/106 (56.4/43.6)	71/62 (53.4/46.6)	66/44 (60.0/40.0)	0.135
Normal range/premature birth	224/19 (92.2/7.8)	126/7 (94.7/5.3)	98/12 (89.1/10.9)	0.001
Healthy birth/complication during birth	225/18 (92.6/7.4)	126/7 (94.7/5.3)	99/11 (90.0/10.0)	0.001
Average birth weight (in kg)	3.36 + 0.47 [1.8–4.8] Me = 3.40, IR = 0.50	3.32 + 0.44 [1.8–4.8] Me = 3.30, IR = 0.50	3.41 + 0.51 [1.8–4.8] Me = 3.45, IR = 0.70	0.116 ^ɫ^
Breastfeeding	229 (94.2)	128 (96.2)	101 (91.8)	0.136
Average period of breastfeeding (in months)	10.27 + 8.70 [0.5–60] Me = 6.0, IR = 6.5	10.47 + 9.23 [0.5–60.0] Me = 6.0, IR = 6.0	10.0 + 8.01 [1.0–48.0] Me = 6.5, IR = 9.0	0.684 ^ɫ^
Feeding with milk formula	14 (5.8)	5 (3.8)	9 (8.2)	0.062
Current diseases	6 (2.5)	1 (0.8)	5 (4.5)	0.117
Diseases in early childhood	15 (6.2)	10 (7.5)	5 (4.5)	0.235
Diseases during lactation	9 (3.7)	2 (1.5)	7 (6.4)	0.435
Medications during lactation	13 (5.3)	7 (5.3)	6 (5.5)	0.688

^ɫ^ Mann–Whitney U-test; * Chi-square test [min–max value]; IR, interquartile range; Me, median. The percentages are calculated in columns.

**Table 8:** General information from birth to early childhood period on children with EH who participated in this study, based on the questionnaire filled in by their parents.

	Total *n* = 31 (%)	Female *n* = 15 (%)	Male *n* = 16 (%)	*p*-Value *
Natural birth	18 (58.1)	10(66.7)	8 (50.0)	0.78
Birth operation	12 (38.7)	4 (26.7)	8 (50.0)	0.113
No answer	1 (3.2)	1 (6.6)	0 (0.0)	
Average birth weight (in kg)	3.42 + 0.35 [3–4.6] Me = 3.3, IR = 0.4	3.33 + 0.22 [3.0–3.7] Me = 3.30, IR = 0.30	3.51 + 0.45 [3.0–4.6] Me = 3.45, IR = 0.62	0.567 ^ɫ^
Breastfeeding	26 (83.8)	12 (80.0)	14 (87.5)	0.325
Average period of breastfeeding (in months)	12.1 + 10.1 [1.6–48], Me = 7.00, IR = 12.5	12.53 + 8.05 [3.0–24.0] Me = 12.00, IR = 14.00	12.11 + 12.39 [1.6–48.0] Me = 6.5, IR = 110.25	0.567 ^ɫ^
Feeding with milk formula	2 (6.5)	0 (0.0)	2 (12.5)	
Current diseases	6 (19.4)	1 (6.6)	5 (31.3)	0.001
Diseases in early childhood	15 (48.4)	10 (66.7)	5 (31.3)	0.03
Diseases during lactation	0 (0.0)	0 (0.0)	0 (0.0)	
Medications during lactation	0 (0.0)	0 (0.0)	0 (0.0)	

^ɫ^ Mann–Whitney U-test; * Chi-square test [min–max value]; IR, interquartile range; Me, median. The percentages are calculated in columns.

## Data Availability

The authors declare that the data in this research are available from the corresponding author upon reasonable request. The data are not publicly available due to ethical.
